# Multiple cardiac metastases from urothelial carcinoma case report

**DOI:** 10.1186/s43044-022-00264-y

**Published:** 2022-04-13

**Authors:** Neil Grech, William Camilleri, Alexander Borg

**Affiliations:** Department of Cardiology, Mater Dei Hospital, Triq id-Donaturi tad-Demm, Msida, MSD2090 Malta

**Keywords:** Case report, Cardiac metastasis, Urothelial carcinoma, Cardiac magnetic resonance imaging

## Abstract

**Background:**

Cardiac metastases are rare and frequently remain undiagnosed due to the absence of clinical signs in the majority of cases. Malignancies found to most commonly metastasise to the heart include lung carcinoma, breast carcinoma and lymphoma, while urothelial carcinoma is a rare cause of cardiac metastasis. The patient presented with pyrexia, a rare presentation of metastatic cardiac involvement. Single metastatic lesions are mainly reported in the literature, while multiple metastatic deposits such as in this case are less common.

**Case presentation:**

A 74-year-old gentleman presented with frequent febrile spikes, a month after undergoing a nephroureterectomy for poorly differentiated urothelial carcinoma. No febrile source was identified, and a computed tomography identified two cardiac lesions. A transthoracic echocardiogram could not detect the cardiac lesions; therefore, cardiac magnetic resonance (CMR) imaging was performed. Three spherical intramyocardial masses were noted at the basal septum, LV apex and the anteromedial papillary muscle. The lesions demonstrated signal characteristics suggestive of cardiac metastases (high fluid content, absence of fat, presence of a surrounding rim of increased extravascular space, absence of deformation within the masses) from the previously resected urothelial carcinoma. The patient was palliated, and he shortly succumbed to his condition.

**Conclusions:**

Urothelial carcinoma is an exceedingly rare cause of cardiac metastasis. CMR is an important imaging modality for localisation and characterisation of suspicious cardiac lesions, aiding in the diagnosis of cardiac metastasis.

## Background

The incidence of cardiac metastasis is highly variable in the literature and ranges from 2.3% to 18.3% [1, 2]. Approximately 90% of cardiac metastases are clinically silent and are often diagnosed post-mortem [1, 3, 4]. Urothelial carcinoma is a rare cause of cardiac metastasis, with only 25 reported cases noted in the literature [5–13]. The majority of these cases have described single myocardial metastases with varying patient presentations. We report a case of three cardiac metastatic lesions involving the myocardium, presenting with frequent febrile spikes, a month after a nephroureterectomy for urothelial carcinoma.

## Case presentation

A 74-year-old gentleman presented to the accident and emergency department complaining of frequent febrile spikes and deterioration in general condition a month after undergoing a nephroureterectomy for urothelial carcinoma. Empirical antibiotics prescribed within the community did not produce any improvement. Examination upon presentation was within normal limits. The patient’s medical and surgical history included hypertension, hypercholesterolaemia, peripheral vascular disease and an uneventful coronary artery bypass surgery five months prior to presentation.

The urothelial malignancy was diagnosed after he initially presented with a few weeks’ history of fever, anorexia and weight loss. Computed tomography (CT) of the thorax, abdomen and pelvis had identified a right upper kidney pole hypodense lesion. Biopsy was performed which reported urothelial carcinoma. Subsequently, the patient underwent a nephroureterectomy which confirmed poorly differentiated, extensively necrotic, Grade 3 (World Health Organisation [WHO] classification) urothelial carcinoma (sarcomatoid variant type) with no lymph node involvement (pathological stage T3N0). The patient had been offered adjuvant chemotherapy due to the high predisposition for distant metastases, but he refused.

**Fig. 1:**
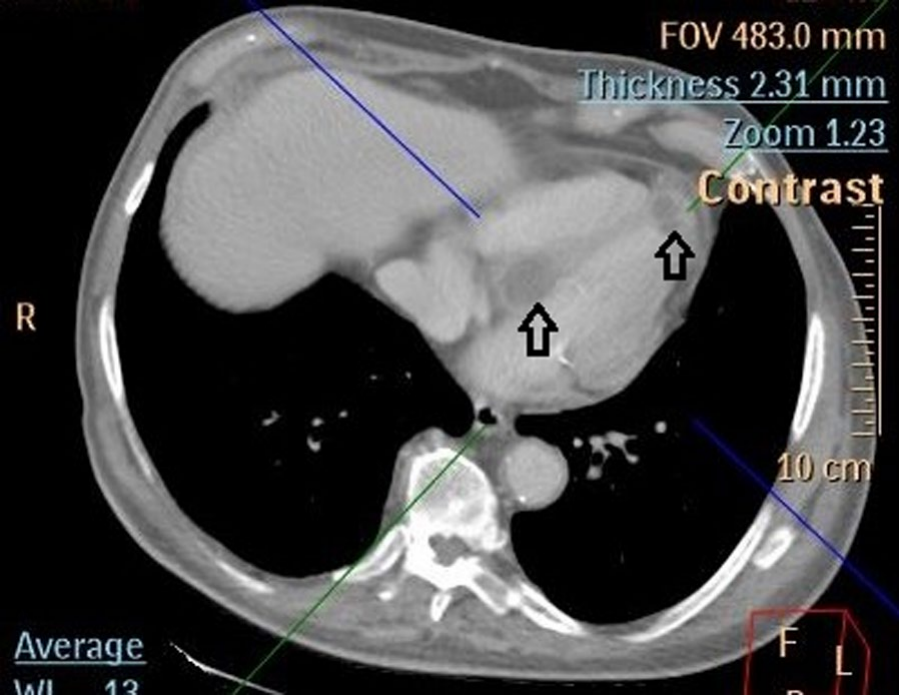
Computerised Tomography identifying a 28 mm lesion at the apex of the left ventricle and another 40 mm lesion in the proximal interventricular wall (indicated by arrows)

During the current admission, no septic focus or causative organism could be identified following multiple blood and urine cultures. Repeat CT of the thorax, abdomen and pelvis was performed, seven weeks following the prior CT, and this identified two new cardiac lesions at the apex of the left ventricle and another in the proximal interventricular wall (Fig. 1). In addition, a peritoneal lesion was noted. Metastatic lesions from the patient’s primary urothelial carcinoma were suspected, particularly since the patient refused post-operative chemotherapy.

**Fig. 2:**
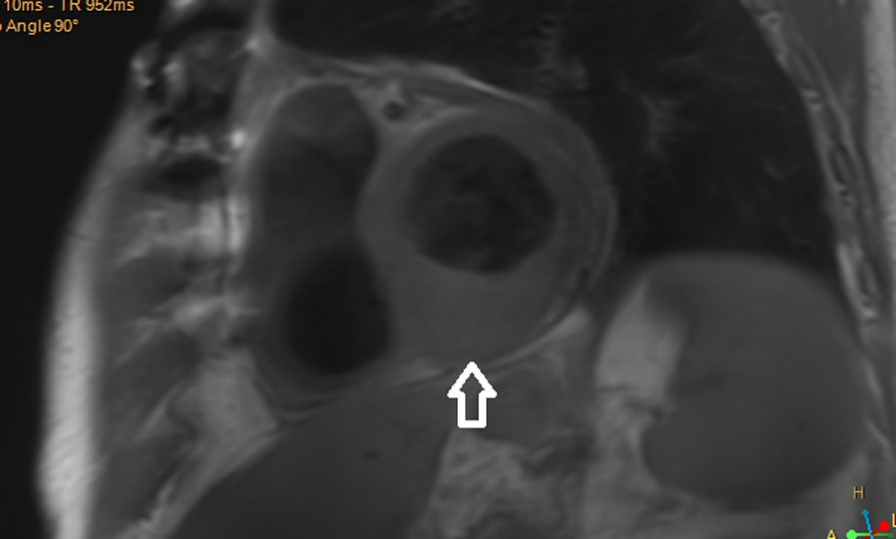
Cardiac Magnetic Resonance Imaging: T1-weighted image in short axis view revealing an isointense lesion at the base of the LV (indicated by arrow)

**Fig. 3:**
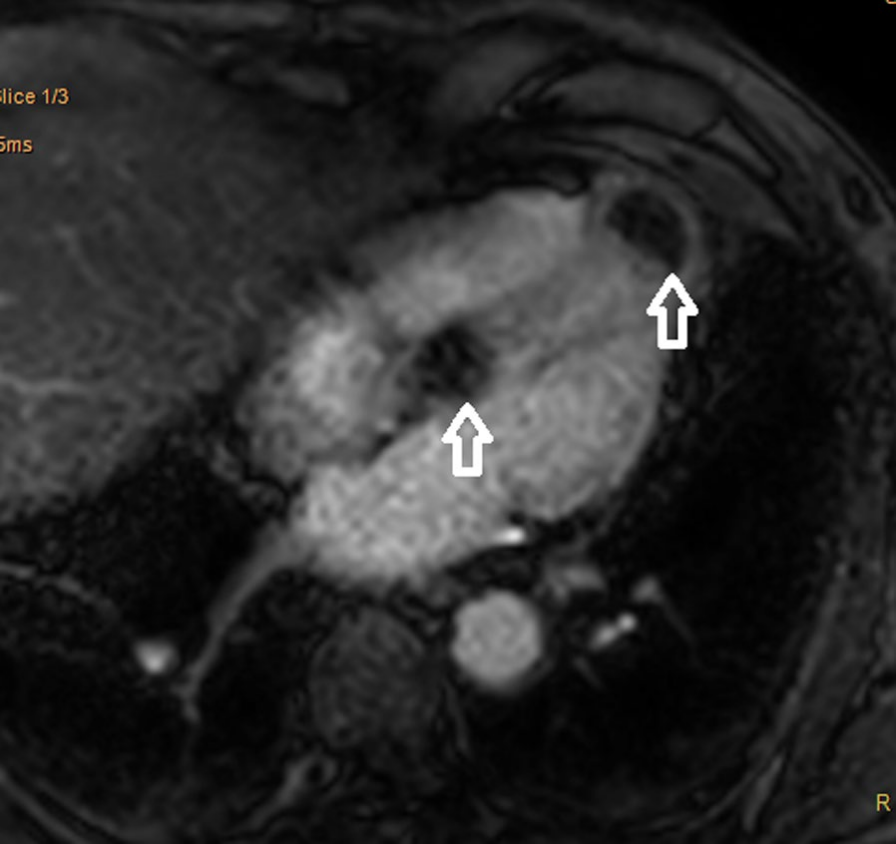
Cardiac Magnetic Resonance Imaging: First pass perfusion in 4-chamber view demonstrating hypoperfusion of the lesions (indicated by arrows) when compared to normal myocardium

**Fig. 4:**
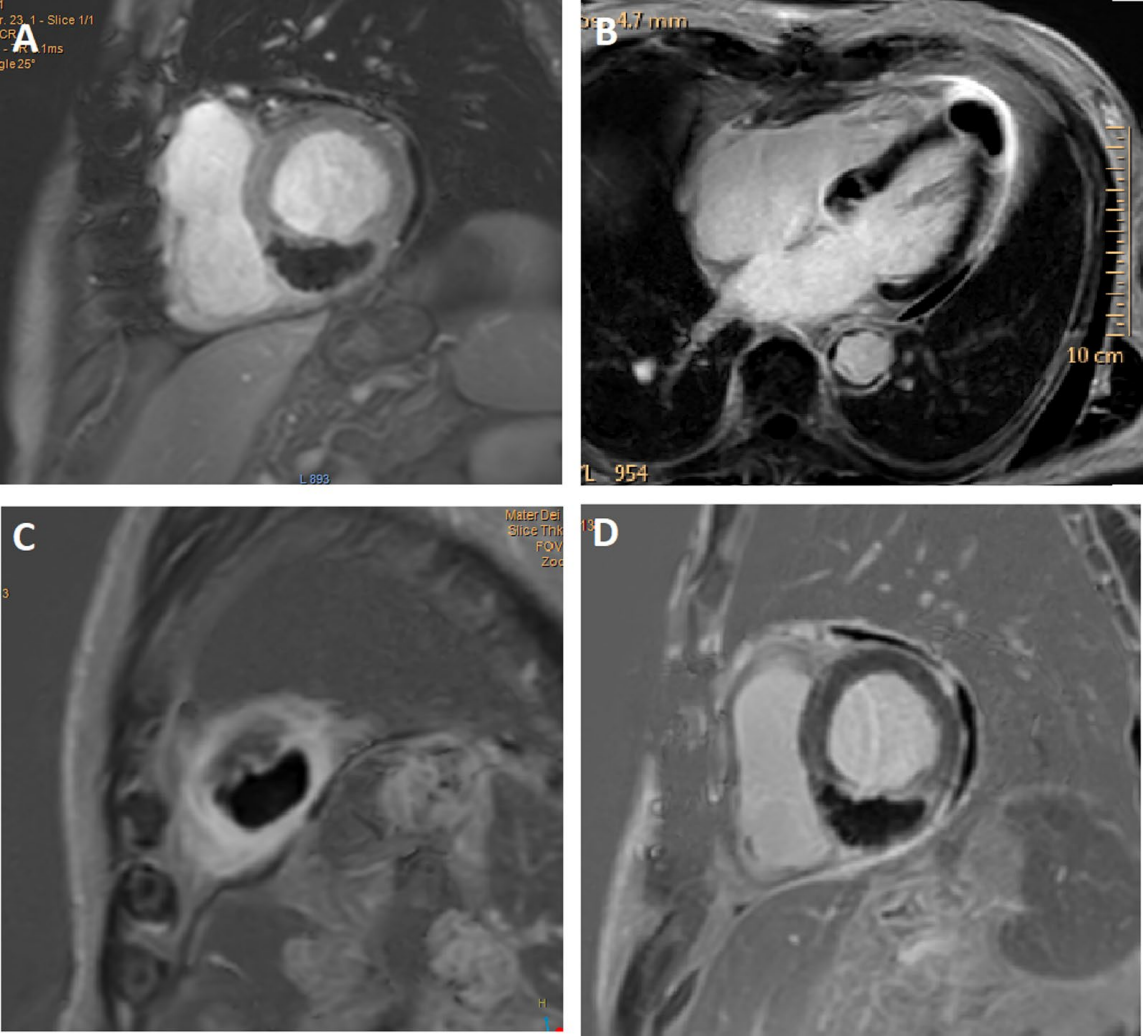
Cardiac Magnetic Resonance Imaging: **A** Early Gadolinium enhancement in short axis view. Late Gadolinium enhancement CMR images in 4-chamber view (**B**) and short axis views (**C**, **D**) revealing two lesions at base of LV apex and base of interventricular septum. Lesions demonstrate no gadolinium enhancement indicating avascular cores

**Fig. 5:**
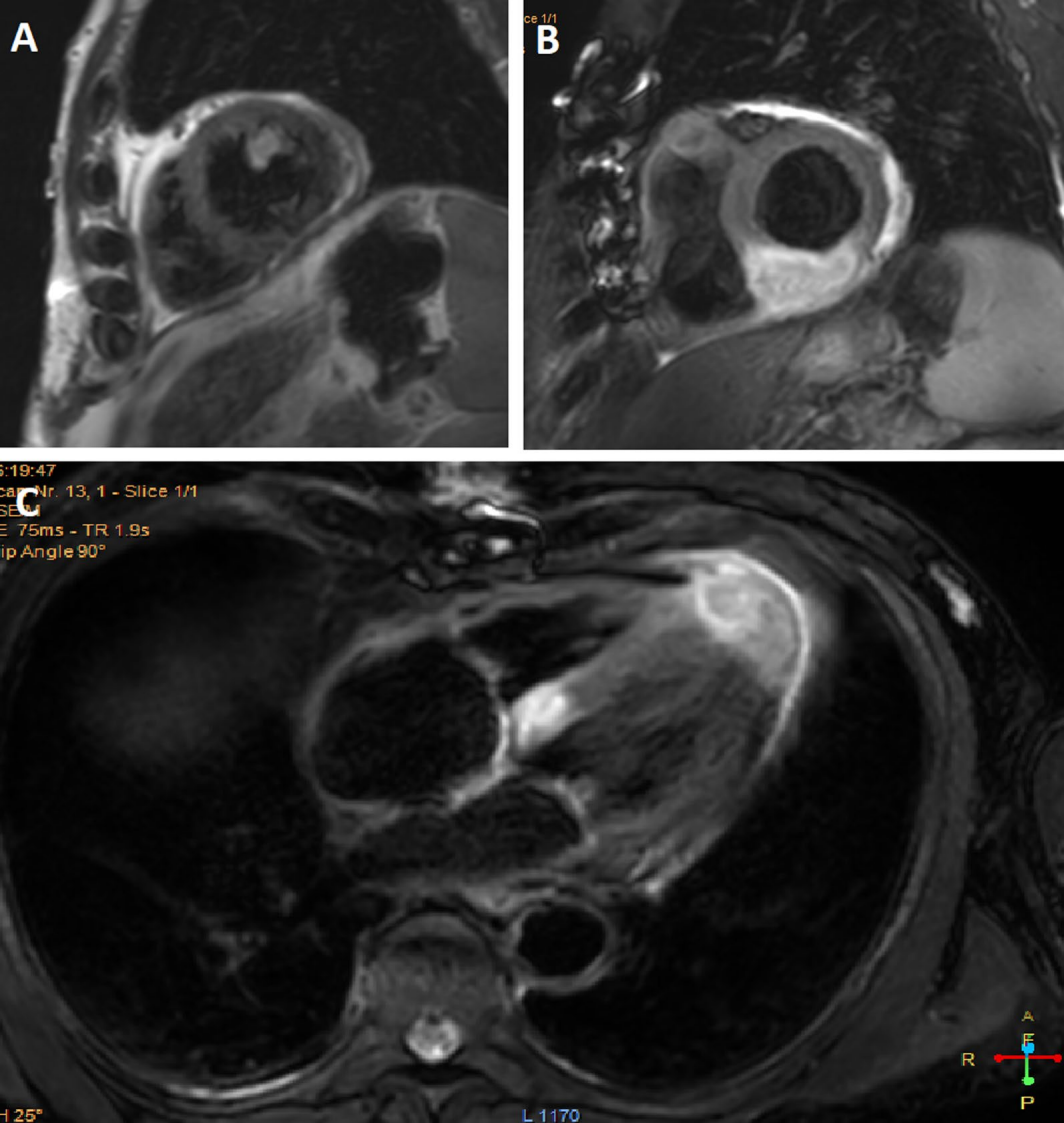
Cardiac Magnetic Resonance Imaging: T2-weighted images in short axis views (**A**, **B**) and 4-chamber view (**C**), identifying three hyperintense lesions (high fluid content), including an anteromedial papillary muscle lesion (**A**)

Transthoracic echocardiography (TTE) could not identify the cardiac lesions. Cardiac magnetic resonance imaging (CMR) was performed which revealed two spherical intramyocardial masses at the basal septum and the left ventricular apex, and another lesion involving the anteromedial papillary muscle. Tissue characterisation of the lesions revealed a high fluid content, absence of fat, presence of a surrounding rim of increased extravascular space, absence of deformation within the masses, and avascular cores (Figs. 2, 3, 4, 5). These features were highly suggestive of intracardiac malignancy, and in this case, they likely represented metastases from the primary urothelial carcinoma.

After multidisciplinary discussion, it was decided to proceed with a biopsy of the peritoneal lesion as it was deemed to be safer and more accessible than the cardiac lesions. An ultrasound-guided biopsy of the abdominal lesion confirmed metastases of the primary urothelial carcinoma. Therefore, given the biopsy result, together with the palliative approach at that stage, and the abundant clinical and imaging evidence favouring the diagnosis of cardiac metastases, a biopsy of the cardiac lesions was not justified.

## Outcome and follow-up

**Fig. 6:**
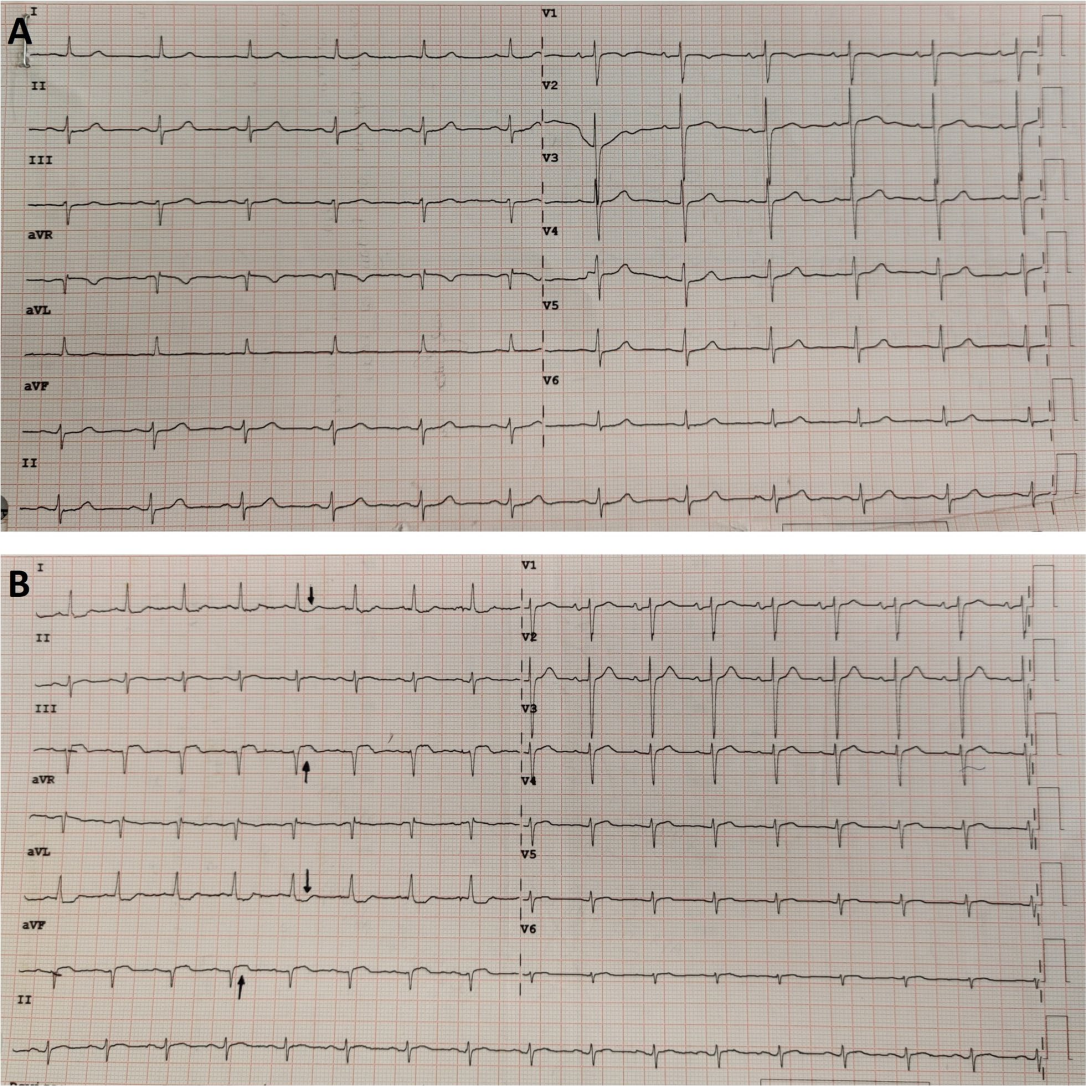
Comparison of 12-lead ECGs taken during the patient’s first presentation (**A**) and last presentation (**B**) to hospital revealing new-onset ST elevations in inferior leads (III and aVF) with Q waves and ST depressions in leads I and aVL (marked with arrows). Paper speed 25 mm/s; voltage 10 mm/mV

The patient was referred to palliative care in view of his poor medical condition. An electrocardiogram revealed new-onset ST elevations in inferior leads (III and aVF) with Q waves and ST depressions in leads I and aVL (Fig. 6). These changes were attributed to the patient’s cardiac metastases. Shortly following admission for presyncope and general deterioration, he suffered an ischaemic cerebrovascular event, eventually passing away within a few days.

## Discussion

Urothelial carcinoma is a rare cause of cardiac metastasis, with only 25 cases reported in the literature. Furthermore, our case is unusual as there were multiple metastases. Fever has also been rarely reported as a presenting complaint.

Cardiac metastases are often clinically silent and are generally discovered during post-mortem examinations, or incidentally on imaging [1, 2]. Bussani et al*.* [2] conducted a study of 18,751 post-mortem examinations where they identified malignancy in 38.8% of cases, among which 9.1% (662 patients) had cardiac metastasis. From the 662 cases, 98.5% had metastases to other sites other than the heart, indicating that cardiac metastasis is usually a feature of widespread disease. The most common malignancies found to metastasise to the heart included lung, followed by breast and lymphoma or leukaemia. Urothelial carcinoma represented only 1.8% of all primary malignancies with cardiac metastasis, making it an exceptionally rare cause for cardiac metastases [2].

Pericardial involvement is the most frequently cited location of cardiac metastasis, followed by the myocardium; while the least common site is the endocardium [14]. Specific to urothelial metastasis, the majority of the cases reported involved the myocardium (19 cases), while the remaining 5 cases involved the pericardium. One case reported a lesion limited to the endocardial layer [5–13]. Similar to our case, only two other cases involved multiple cardiac metastatic lesions from a urothelial primary; Ueda et al*.* [6] reported a case of right and left myocardial metastases, while Sugimoto et al*.* [9] reported multiple myocardial metastatic nodules.

Patients with cardiac metastasis are often asymptomatic and are primarily noted on post-mortem examination, as mentioned above. If symptomatic, the symptoms reported by patients are often ambiguous. Symptoms of other organ metastases may be prevalent by the time metastasis to the heart occurs, explaining why cardiac metastases often remain undiagnosed. However, cardiac-specific symptoms may occur depending on the site of the metastatic lesions. Patients with pericardial metastasis may present with a pericarditis-like picture, including the development of a pericardial effusion and possibly tamponade [7, 9, 15]. Myocardial involvement may result in arrhythmic complications including life-threatening ventricular fibrillation or complete atrioventricular block [6, 16, 17]. Myocardial involvement may also compromise cardiac output mimicking congestive heart failure. Metastasis to the endocardium may cause left or right outflow obstruction with cardiogenic shock [17]. Any of the described symptoms in a patient diagnosed with malignancy should raise concerns for possible cardiac metastasis. Among the 25 reported cases of cardiac metastases from urothelial carcinoma, many reported non-specific symptoms such as fatigue, cough, and weight loss, among others. Shortness of breath was also reported which may have been secondary to tamponade, a compromised cardiac output or lung metastasis. Among these 25 patients, only 2 cases reported fever as one of the presenting symptoms, similar to our case [7, 8].

Imaging modalities are the main investigations aiding in the diagnosis of cardiac metastasis, however, an electrocardiogram (ECG) may also contribute by revealing low voltage complexes (due to a pericardial effusion), arrhythmias, or nonspecific ST-T wave abnormalities; the latter being reported in our case [18]. Chest x-ray and echocardiography may reveal a pericardial effusion, while echocardiography may also identify cardiac masses [17]. CT, CMR or positron emission tomography-computed tomography (PET-CT) offer more definite imaging to localise and characterise cardiac masses while excluding differentials such as intracardiac thrombus, vegetations, and benign lesions. Our case offers an insight into the role of CMR in cardiac metastasis diagnosis; an imaging modality that was not frequently used in the literature of similar cases.

CMR protocols vary depending on the institution and involve different sequences which must be tailored towards the suspected cardiac pathology. Commonly used sequences include black-blood T1-weighted sequences (with and without fat saturation and before and after contrast enhancement), black-blood T2-weighted imaging, cine imaging with a steady-state free-precession (SSFP) sequence and early and late gadolinium enhancement (LGE) imaging [19]. All these sequences were included in the reported case as part of the institution’s tumour protocol. Characteristics indicative of cardiac metastasis include smooth and spherical lesions, low or isointense T1-weighted images, hyperintense T2-weighted (owing to the high fluid content of malignant lesions), heterogenous LGE, no signal change on fat suppression sequence (absence of fat), presence of pericardial effusion (likely haemorrhagic), and haemorrhage within the mass [19–21]. The tumours in our case demonstrated the above-mentioned characteristics (Figs. 2, 3, 4, 5). Although a clinical diagnosis of cardiac metastasis can often be made on imaging, tissue histology remains the investigation of choice for diagnosis [15]. Cardiac biopsy and treatment of the patient’s condition in the case were deemed ineffectual due to his poor condition.

## Conclusions

Urothelial carcinoma is a rare cause of cardiac metastasis, with only two reported cases of multiple metastatic lesions, and another two cases presenting with fever, analogous to our case. The myocardium is a common site for metastasis to the heart from urothelial carcinoma. CMR imaging is an important tool as it offers detailed characterisation and localisation, aiding in the diagnosis of cardiac metastasis.

## Data Availability

Data sharing is not applicable to this article as no datasets were generated or analysed during the current study.

## Abbreviations

CMR: Cardiac magnetic resonance
CT: Computed tomography
TTE: Transthoracic echocardiography
ECG: Electrocardiogram
PET-CT: Positron emission tomography-computed tomography
LGE: Late gadolinium enhancement
SSFP: Steady-state free-precession
